# A high-throughput pipeline for phenotyping, object detection and quantification of leaf trichomes

**DOI:** 10.1007/s00122-025-04967-z

**Published:** 2025-07-21

**Authors:** Andrea González-Muñoz, Dai-Jie Wu, Ana B. Perera-Rodríguez, Mohamed Rekik, Silvio Giancola, Brande B. H. Wulff, Catherine Gardener

**Affiliations:** 1 Plant Science Program, Biological and Environmental Science and Engineering Division, King Abdullah University of Science and Technology (KAUST), Thuwal, Saudi Arabia; 2 Thya Technology, KAUST, Thuwal, Saudi Arabia

## Abstract

****Key message**:**

We developed a new high-throughput device and AI image detection model capable of rapidly collecting phenotype data for a population of wild grass, facilitating identification of genomic regions associated with trichome density.

**Abstract:**

Access to increasing amounts of high-quality genomic sequence data for many plant species is allowing for faster, more accurate gene identification. To maximize the use of this sequence data for association genetics, gene discovery, and validation, it must be coupled with phenotype data. However, phenotype data acquisition can represent a bottleneck in studies requiring many datapoints, such as large diversity panels for genome-wide association studies. Here we developed a portable handheld imaging device—the Tricocam—and method for image capture and semi-automated quantification of leaf edge trichomes in a grass species. Trichomes have been implicated in abiotic and biotic stress tolerance in grasses, but so far, no trichome genes have been cloned in this plant family. We also refined and implemented the AI detection processes underpinning the web-based image quantification platform from Thya Technology® to rapidly quantify leaf edge trichomes. We used the phenotype acquisition method in the wild wheat progenitor *Aegilops tauschii* in combination with *k*-mer-based Genome-Wide Association Study to validate a trichome-controlling genomic region on chromosome arm 4DL and discover a new one on 4DS. By making the Tricocam 3D print design and AI visual detection model public, we aim to deliver useful resources for the plant science community to use or adapt for other large-scale phenotyping projects on diversity panels.

**Supplementary Information:**

The online version contains supplementary material available at 10.1007/s00122-025-04967-z.

## Introduction

Trichomes are hair-like structures on the aerial surfaces of plants that play an important role in protection against herbivores, excessive radiation, water stress, and even in facilitating pollination (Wang et al. 2021; Lustofin et al. 2023). Trichomes can be glandular or non-glandular depending on their structure and role. Glandular trichomes produce and secrete substances, whereas non-glandular trichomes are primarily involved in physical protection (Han et al. 2022). In grass species, the role of non-glandular trichomes is not well defined, but presumably they play equally important roles in abiotic and biotic stress tolerance (Kim 2019; Pshenichnikova et al. 2019; Batyrshina et al. 2020). Trichomes in these species can have positive or negative effects on plant fitness depending on environmental conditions, so wild grass species are subjected to opposing selective pressures for this trait (Karkkäinen et al. 2004; Singh et al. 2021; Sun et al. 2024). This has led to a high degree of diversity in leaf trichome number in populations of wheat wild relatives (Luo et al. 2022; Mahjoob et al. 2022).

Trichome formation and development is therefore highly regulated through genetic and environmental factors. Genetic regulation is attributed to a complex network of transcription factors exerting positive or negative control and exhibiting functional redundancy, as has been demonstrated in detail in Arabidopsis, rice, tomato, cotton, cucumber, and tobacco (reviewed in Han et al. 2022 and Kabir et al. 2024). Transcription factor families that regulate trichome development in these species include MYB family proteins, C2H2 zinc finger proteins, HD-ZIP-type proteins, and basic helix-loop-helix (bHLH)-type proteins (reviewed in Khan et al. 2021). In addition to these genetic factors, hormone signaling and water and light conditions are also key players in the regulatory network for trichome development (Kabir et al. 2024; Yan et al. 2017).

In the grasses, diversity panels of wild wheat species can act as reservoirs for genetic variation for traits conferring environmental resilience (Gaurav et al. 2022). The wild wheat *Aegilops tauschii*, the D genome donor of bread wheat, has preserved such genetic diversity that has been lost in cultivated wheat due to bottlenecks imposed by polyploidization, domestication, and selective breeding. *Ae. tauschii* exhibits high leaf trichome density variation, and accessions originating from eastern habitats differ in trichome density from germplasm from western habitats (Morihiro & Takumi 2010). High leaf trichome density is reported predominantly in Transcaucasus accessions and those from northern Iran, while low leaf trichome density is observed mostly in *Ae. tauschii* accessions from Afghanistan and Pakistan (Morihiro & Takumi 2010).

The genetic control of trichome development in wheat and its wild relatives has not been extensively studied beyond the identification of major quantitative loci (QTL). In *Ae. tauschii*, marker-trait associations for trichome variation were identified on chromosomes 2D, 3D, 5D, 7D (Mahjoob et al. 2022) and 4DL (QTL designation QHl.ipk-4D) (Dobrovolskaya et al. 2007; Gaurav et al. 2022). In bread wheat, trichomes presence or absence has been attributed to variation in the *Hg2* locus on chromosome 2B, *Hl1* on 4B, *Hl2* on 7B, and *Hg* on 1A (Dobrovolskaya et al. 2007; Chen et al. 2021; Luo et al. 2016, 2022; Wu et al. 2021). Additional QTL have been identified in other Triticeae species, such as barley (*Hs* locus on chromosome 4HL) (Saade et al. 2017) and rye (*Hp1* locus on chromosome 5RL, homeologous to *Hs* in barley) (Chang 1975; Korzun et al. 1999). While there are many QTL identified for trichome variation in wheat and many studies implicate these QTL in various types of biotic and abiotic stress tolerance (David et al. 2017), until these genes are cloned, we will not be able to generate true isogenic lines that allow unambiguous assignment of trichome variation to these biotic and abiotic stress phenotypes (Chen et al. 2021).

Genome-wide association studies (GWAS) can identify linked genomic regions containing genes or regulatory elements underlying the genetic control of agronomic traits of interest like trichomes (Lemay et al. 2023). In GWAS, single nucleotide polymorphisms (SNPs) are commonly used as genetic markers for detecting marker-trait associations, relying on the linkage between a SNP and the true causal variant. However, limitations of this SNP-based approach include restricted and possibly inaccurate genotyping because of the dependence on a single reference genome for SNP calling, since the reference could be lacking the causal variant or associated genomic region (Karikari et al. 2023). Furthermore, SNPs can fail to detect associations, and it can be difficult to accurately pinpoint the causal variant. The advent of *k-*mer-based GWAS (*k*GWAS) has allowed for detection of genetic elements such as large structural variations, which cannot be easily detected through traditional SNP-based GWAS (Karikari et al. 2023). Several studies have demonstrated the use of *k*-mers to capture additional genetic variants unaccounted for by SNPs and to discover associations arising from structural variation (Jaegle et al. 2024; Lemay et al. 2023). GWAS analyses require phenotype data from a population of genetically different individuals. In many cases, collecting phenotype data is time-consuming and largely dependent on human input, thus representing a bottleneck in studies of this type. To overcome this barrier, many automated or semi-automated plant phenotyping systems have been devised and are available for use in plant growth facilities (Li et al. 2021; Zhang et al. 2023). However, these systems often require installation of complex sensors and operation by skilled technicians. They can also be patent-protected and expensive or difficult to implement. Therefore, there is a need for lower-technology innovation in the form of smaller, cheaper devices, which can be tailored for phenotype acquisition according to the research project.

To keep up with data acquired by such high-throughput devices, phenotypic images must be coupled with software facilitating automated analysis. Automated image detection models have been developed and implemented in a wide variety of contexts, including the development of Faster R-CNN to detect and count germinated Striga seeds (Braguy et al. 2021), an Ilastik-Fiji tandem machine learning approach to count trichomes in Arabidopsis (Garcia et al. 2022), HairNet2 for quantifying cotton leaf hairiness (Farazi et al. 2024), and TrichomeYOLO for maize trichome counting (Xu et al. 2023). Similarly, YOLO object detection models have demonstrated efficacy in multi-class weed detection for cotton fields, leveraging large-scale annotated datasets to achieve high detection accuracy in real time (Dang et al. 2023). In rice fields, a novel multi-scale feature enhanced DETR model (RMS-DETR) was recently proposed to address the challenges of identifying small, occluded, and densely distributed weeds, achieving significant improvements in precision and recognition accuracy (Guo et al. 2024). These advancements highlight the potential of deep learning models like Faster R-CNN, YOLO, and RMS-DETR to transform plant phenotyping and management in complex agricultural environments.

Another crucial parameter in plant genotype-to-phenotype correlation studies that has shown drastic changes in recent years is the cost of sequencing (Li et al. 2021). Recently, a genomic resource of 920 Illumina short-read-sequenced *Ae. tauschii* accessions, along with 46 genomes assembled from PacBio high-fidelity long reads, has been made available (Cavalet-Giorsa et al. 2024). In this study, we leverage a simple, high-throughput image acquisition and trichome detection method with *k*-mer-based GWAS as a proof-of-concept to streamline genotype–phenotype correlation efforts in *Aegilops tauschii*. We aim to demonstrate how the time and manual input for GWAS-based association analyses can be significantly reduced through the development and implementation of a leaf trichome phenotyping method based on a low-cost, high-throughput imaging device and automated image detection.

## Materials and methods

### Plant germplasm and growth conditions

*Aegilops tauschii* germplasm previously described in Cavalet-Giorsa et al., (2024) was used for this study. *Ae. tauschii* has a population structure comprising three genetically distinct lineages designated as Lineage 1 (L1), Lineage 2 (L2), and Lineage 3 (L3) (Gaurav et al. 2022). We analyzed 616 accessions, including 358 L1, 203 L2, and 6 L3 accessions. Four plants per accession were germinated as follows: Seeds were imbibed on filter paper and kept at 4 °C for 3 days in the dark, followed by 3 days in the light, at room temperature on a laboratory bench. They were transferred to soil in individual 9 × 9 cm pots, 18 pots per tray. Plants were grown until the 3-leaf unfurled stage (Tottman 1987) under speed breeding conditions (22-h day at 22° C, 6-h night at 18 °C, no controlled humidity, and 70% red light, 70% blue light, 70% white light, 10% infrared) (Watson et al. 2018) in an air-conditioned greenhouse supplemented with light from ELIXIA LX602C Lamps (Heliospectra, The Netherlands) in KAUST.

### Statistical analyses and broad-sense heritability estimate for leaf edge trichome density in *Aegilops tauschii*

The phenotyping of the full panel of 616 accessions was conducted once in a greenhouse environment, with measurements taken for two leaves of 3–4 plants per accession. However, to estimate broad-sense heritability of leaf edge trichome density, we selected 41 accessions with varying trichome density scores and grew them in replicated experiments in three controlled growth environments (Supplementary Table 2). The germination conditions and planting arrangements in the pots and trays were conducted as described above. We scored the leaves of three seedlings per accession in each environment. We performed linear mixed-effects modeling using the lmerTest and nlme R packages (Kuznetsova et al. 2017; Pinheiro et al. 2023). To estimate broad-sense heritability (H^2^), we modeled genotype, environment, and their interaction as random effects using lme. To test fixed effects and perform type III ANOVA, we used lmerTest, treating genotype, environment, and their interaction as fixed effects.

Broad-sense heritability (H^2^) was estimated as given by the equation below:

$$H^{2} = \frac{{A_{V} }}{{A_{V} + E_{V} + \left( {\frac{{GxE_{V} }}{{n_{{{\text{ENV}}}} }}} \right) + e}}$$

where A_V_ is the additive genetic variance, E_V_ is the environmental variance, G x E_V_ is the genotype-by-environment variance, *n*_ENV_ is the number of environments, and *e* is the residual variance.

### Association genetics analysis in *Aegilops tauschii*

We used the *k*-mer-based GWAS (*k*GWAS) pipeline described by Gaurav et al. (2022) (https://github.com/wheatgenetics/owwc/tree/master/kGWAS).

The *k*-mer presence/absence matrix and high-quality *Ae. tauschii* genome assembly of accession TOWWC0023 for the association mapping were published by Cavalet-Giorsa et al. (2024) and downloaded from DRYAD (Cavalet-Giorsa et al. 2024). Leaf edge trichome density phenotype data for 140 non-redundant *Ae. tauschii* accessions (Supplementary Table 1) were used for analysis together with the leaf edge trichome density phenotype data previously published by Gaurav et al. (2022) for 116 non-redundant Lineage 2 accessions. The 116 accessions from Gaurav et al. (2022) are a subset of the 140 Lineage 2 accessions that were analyzed in this study (Fig. S1). Thus, we were able to compare *k*GWAS results for the same set of accessions phenotyped with two different methods.

### Data visualization

The histogram plots for leaf edge trichome density in *Aegilops tauschii* were generated using Python seaborn library v0.11.2 (Waskom 2021) in Jupyter notebook (Kluyver et al. 2016). We generated Q-Q plots for the -log10 (*p* values) of the significantly associated *k*-mers from the *k*GWAS using the qqman R package (Turner 2018). All R packages used in this study were implemented in RStudio version 2023.03.1 + 446 with R4.3.0 (R Core Team 2023). The Manhattan plots were generated using the gwas_plot.py code from the *k*GWAS pipeline described by Gaurav et al. (2022). (https://github.com/wheatgenetics/owwc/tree/master/kGWAS).

## Results

### A 3D-Printed imaging system for non-destructive leaf trichome capture

**Fig. 1:**
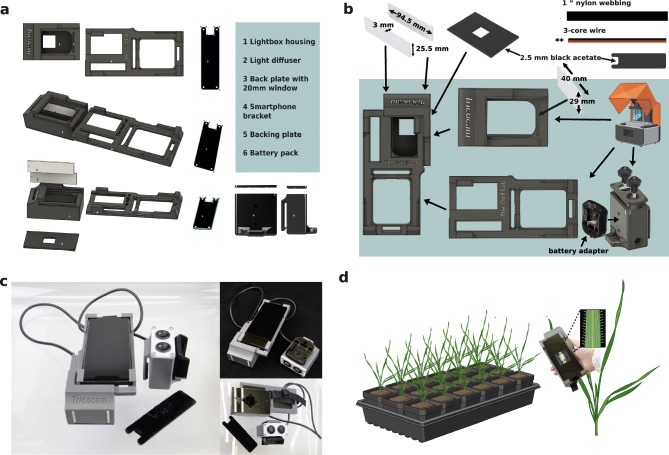
High-throughput and non-destructive leaf imaging in *Aegilops tauschii* using the Tricocam. **a** Diagram of device structure. **b** Tricocam assembly process. **c** Assembled Tricocam. **d** Phenotyping process with Tricocam

In a previous study, a non-automated method was utilized to measure leaf edge trichome density using manual imaging with a camera (Gaurav et al. 2022). To refine this method, we leveraged the availability of high-resolution cameras in smartphones and 3D printing systems which can create made-to-measure components with little specialist training. To maximize the utility of this device, we designed it to capture leaf images from plants non-destructively, allowing plants to be used for other experimental purposes such as infection or for phenotyping at a later growth stage. The portable handheld device comprises a small lightbox lined bilaterally with LED strips, a holder for a smartphone, and a back plate window with built-in scale for easy capture of leaf surface images. A battery pack, compatible with a standard 12 V drill battery, allows for prolonged usage, while a backing plate facilitates maneuvering leaves into position for imaging (Fig. 1a).

### Lightbox printing and assembly

3D designs were conceived using Autodesk Fusion 360 design software (https://www.autodesk.com/ae/products/fusion-360/overview). The lightbox, phone holder, and battery pack were printed separately using a Form 3/3 + Desktop resin 3D printer (Form Labs, USA) with standard gray resin in the Prototyping Laboratory at KAUST (Saudi Arabia) (Fig. 1b). For assembly of the lightbox, two pieces of 3-mm translucent white acetate were cut using a laser cutter to 94.5 × 25.5 mm. One additional piece was cut to 29 × 40 mm and one to 70 × 35 mm, including 4 mm rounded corner radius and holes for switches, as detailed in 3D Autodesk and laser cut.dxf files (Supplementary data files 1–3). Strips of Onforu White LED lights (amazon.co.uk) were cut to size, including 2 strips of 3 LEDs on each side, and attached inside the lightbox. The back plate for the lightbox was cut by laser from 2.5 mm black acetate, to include a hole of 24.5 mm. Black self-adhesive IR Flock Sheet backing fabric (the-black-market.com) was attached to the side of the acetate facing the lightbox. Once the backing fabric was attached, the hole size was reduced to 20 mm by the fabric, serving as a scale in the captured images. The back plate was fixed in place with heavy-duty double-sided tape. A phone holder was designed and printed as above for a Samsung Galaxy S21 Ultra 5G, to include struts to affix a strap. Dimensions of the Samsung Galaxy S21 Ultra 5G are 164 L × 75 W × 11 D (maximum depth at camera) mm. The phone holder can be adjusted to fit other Samsung phone models by using the “scale” feature in Autodesk 360 to adjust the phone holder and lightbox together to the relative size of the desired smart phone. Other brands of phone may also work, where the camera placement is the same as Samsung models. Multiple tutorials are available online for editing 3D models using Autodesk as well as altering the files for laser cutting.

To finish the Tricocam device, a strap was made using 1″ black nylon webbing and hand-sewed to the back of the phone holder. Finally, a battery pack was designed and customized to hold a 12 V 3.0 Ah Li-ion drill battery with an IRONFACE Adapter for Dewalt 12v Max Battery power Connector (desertcart.com.sa) and two 23-mm Mini-Rocker switches (amazon.sa) to allow for mono- or bilateral illumination of subject leaves by the lightbox. Wires were threaded through a hole in the back of the lightbox and attached to the battery pack using 1 m length of wrapped 3-core wire. This allows for the battery pack to be attached to external clothing or pockets for ease of use. Switches were inserted into the battery pack top plate, wired in, and attached using screws. The acetate strips were slid in to cover the LEDs and diffuse the light (Fig. 1b). The additional backing plate to isolate the leaves for imaging was laser cut to 120 × 34 mm, with a box of 15 × 15 mm removed and covered with adhesive black fabric. The assembled Tricocam can be seen in Fig. 1c.

To collect standardized images of leaf surfaces, leaves of 2- to 3-week-old (3 leaves unfolded stage (Tottman 1987)) *Ae. tauschii* seedlings were imaged. The backing plate was used to capture the leaf abaxial side up and manually pressed to the back of the lightbox for each leaf. After setting the smartphone to 0.9 × zoom and focusing the camera on the leaf surface, the photograph was taken (Fig. 1d). Two leaves per plant were imaged for 3–4 plants per accession, which took around 10–15 s per plant, allowing phenotyping of approximately 240 plants per hour to be carried out in a non-destructive manner.

### Image processing for AI detection

To enable fast image processing and leaf edge trichome counting, we used a new web-based AI training and image detection platform from Thya Technology, Thuwal, Saudi Arabia. The image detection process was most effective when working with aligned, cropped images in the same orientation. Therefore, images were sorted into horizontal and vertical orientations and cropped to squares including only the viewing window (Fig. S2). In total, 4477 images were collected from the panel of *Ae. tauschii* accessions. The coordinates of the edges of each image were determined using Image J and used as input for the cropping code. All images were then aligned and cropped before training and inputting into the platform for detection.

### Development of the object detection algorithm and augmentation methods

**Fig. 2:**
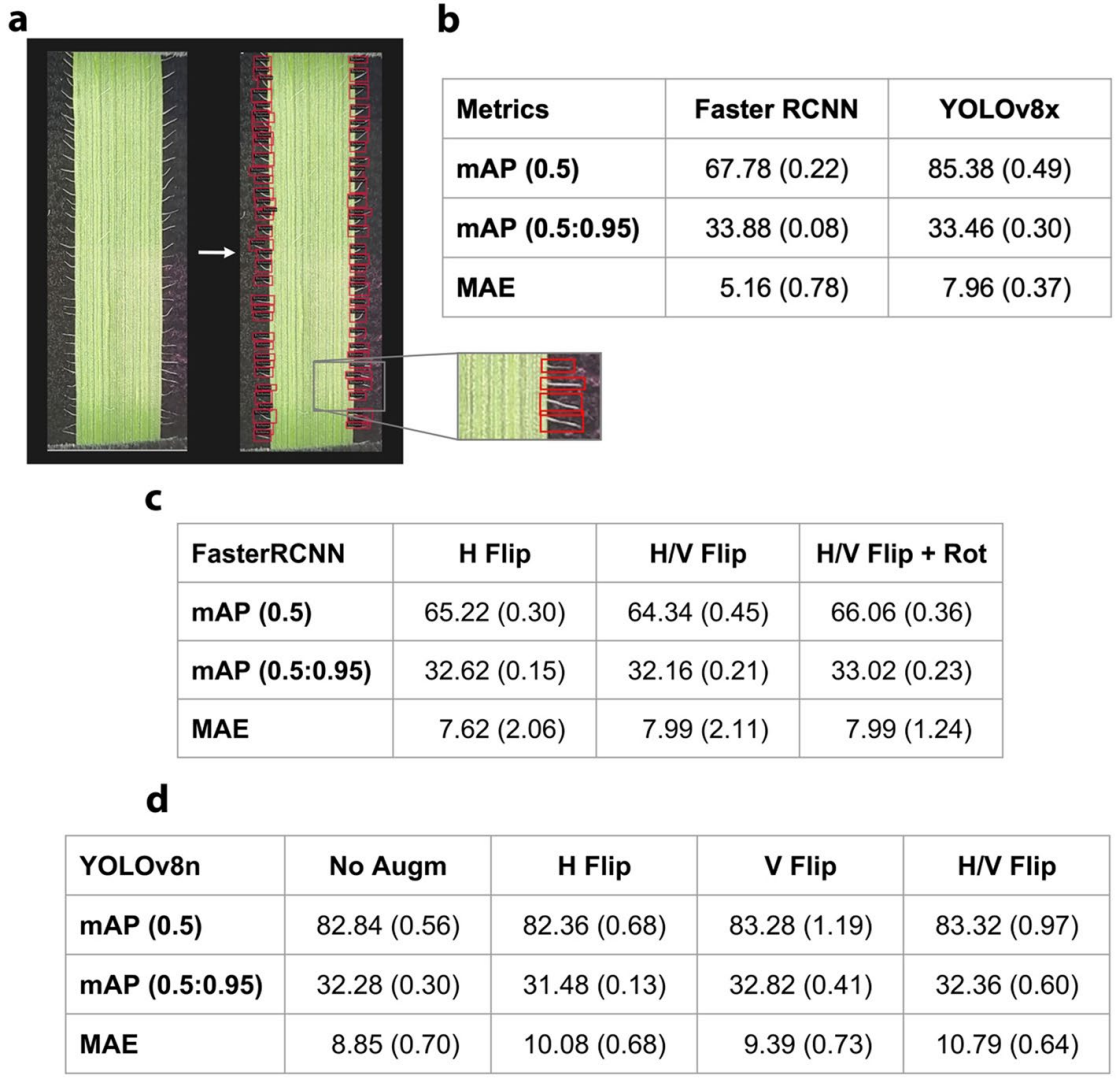
Development of object detection algorithm and augmentation methods. **a** Automated detection of leaf edge trichomes in *Aegilops tauschii*. Detected trichomes are indicated by red boxes. **b** Comparative analysis of the two different AI detection models, Faster R-CNN and YOLOv8. mAP(0.5) considers a single threshold for the IoU set to 0.5, mAP(0.5:0.95) averages over 10 stricter IoU threshold ranging from 0.5 to 0.95, with intermediate steps of 0.05. **c** Analysis of effects of image augmentation on detection accuracy with Faster R-CNN. **d** Comparative analysis of image augmentation with YOLOv8

To train the algorithm for image detection, trichomes in a subset of cropped images were identified by eye and bound manually by a box (Fig. 2a). The model was retrained multiple times, each with a gradually increasing number of annotated photographs. In the final model, more images with higher density of trichomes were included for training as these proved the most difficult for accurate detection. The accuracy was also improved by including a leaf detection box, thus restricting trichome detection to the leaf area. This reduced the number of false positive trichome detections, which were primarily small particles on the backing plate far away from the leaf surface. Finally, a set of 84 images across the spectrum of trichome density was used to train the model, which achieved a counting error of 5.16 trichomes per image compared to the same dataset when manually assessed by eye. This is comparable to the accuracy obtained in the previous study using the same AI model (Braguy et al. 2021).

A prediction is classified as a True Positive (TP) if its Intersection over Union (IoU) with a ground truth (GT) bounding box exceeds a predefined threshold. Predictions that do not match any GT box are classified as False Positives (FP), while GT boxes with no matching predictions are considered False Negatives (FN). Using these classifications, recall is calculated as Recall = TP/(TP + FN), representing the proportion of GT boxes that are correctly predicted, and precision is defined as Precision = TP/(TP + FP), measuring the proportion of correct predictions among all predicted bounding boxes.

We evaluated the model’s accuracy using two key metrics, the mean Average Precision (mAP) and the Mean Average Error (MAE). The mean Average Precision (mAP) summarizes the model’s performance by combining precision and recall for each class. For this work, which focuses on a single class (trichome), the mAP is equivalent to the Average Precision (AP), calculated as the area under the precision–recall curve as the detection confidence score varies. This metric provides a comprehensive evaluation of the model’s ability to detect and localize objects within the single class of interest. The mAP is declined in 2 versions, the mAP@0.5 which considers a single threshold for the IoU set to 0.5, and a mAP@0.5:0.95 that averages over 10 stricter IoU threshold ranging from 0.5 to 0.95, with intermediate steps of 0.05.

Additionally, the accuracy of quantifying the true number of image features detected by the algorithm was measured using the mean average error (MAE). MAE quantifies the average difference between the number of detected objects (N_d_) and the actual number of ground truth objects (N_gt_) across all images, providing a direct measure of the model’s ability to count features accurately. It is calculated as the sum of the absolute differences between the detected and ground truth counts for each image, divided by the total number of images. Mathematically, this is expressed as:

$${\text{MAE}} = \frac{1}{n}\mathop \sum \limits_{i = 1}^{n} |N_{d,i} - N_{{{\text{gt}},i}} |$$

where n is the total number of images, $N_{d,i}$ is the number of objects detected in image i, and $N_{{{\text{gt}}, i}}$ is the number of ground truth objects in image i.

The object detection algorithm employed by the Thya Technology platform uses the Faster R-CNN supervised deep learning algorithm with high prediction accuracy (Braguy et al. 2021). Moreover, we investigated the more recent object detection algorithms YOLOv8, known for its higher accuracy, faster inference speed, and capability of edge deployment on smartphone devices. In general, this alternative model was more accurate at predicting the bounding box of each trichome, but no increase in quantification accuracy was observed (Fig. 2b).

YOLOv8 achieved a mAP of 85.38%, significantly outperforming Faster R-CNN, which attained a mAP of 67.78%. This improvement is attributed to YOLOv8’s advanced architecture, which integrates multi-scale feature fusion and real-time inference capabilities, enabling more precise localization of trichomes in our images.

However, this enhanced detection accuracy did not translate to improved quantification accuracy. Faster R-CNN achieved a lower MAE of 5.16, indicating better alignment with the true count of trichomes. In contrast, YOLOv8x exhibited a higher MAE of 7.96, reflecting a tendency to over- or under-detect trichomes, especially in high-density regions. This discrepancy suggests that YOLOv8x, while adept at precisely determining the location of individual trichomes, struggles with distinguishing overlapping structures, so is less effective at determining the number of true trichomes in an image compared with the R-CNN model.

We further explored the potential of image augmentation to further enhance model performance. To that end, the model was trained using images that had been flipped, rotated, or both to assess whether improvements were reflected in mAP or MAE scores (Fig. 2c). Improvements were observed for mAP, but the absolute error of detection was not improved. Finally, the model was trained with YOLOv8 and data augmentation, with the same result (Fig. 2d).

### Identification of genomic regions associated with leaf edge trichome density using a large-scale dataset in *Aegilops tauschii*

**Fig. 3:**
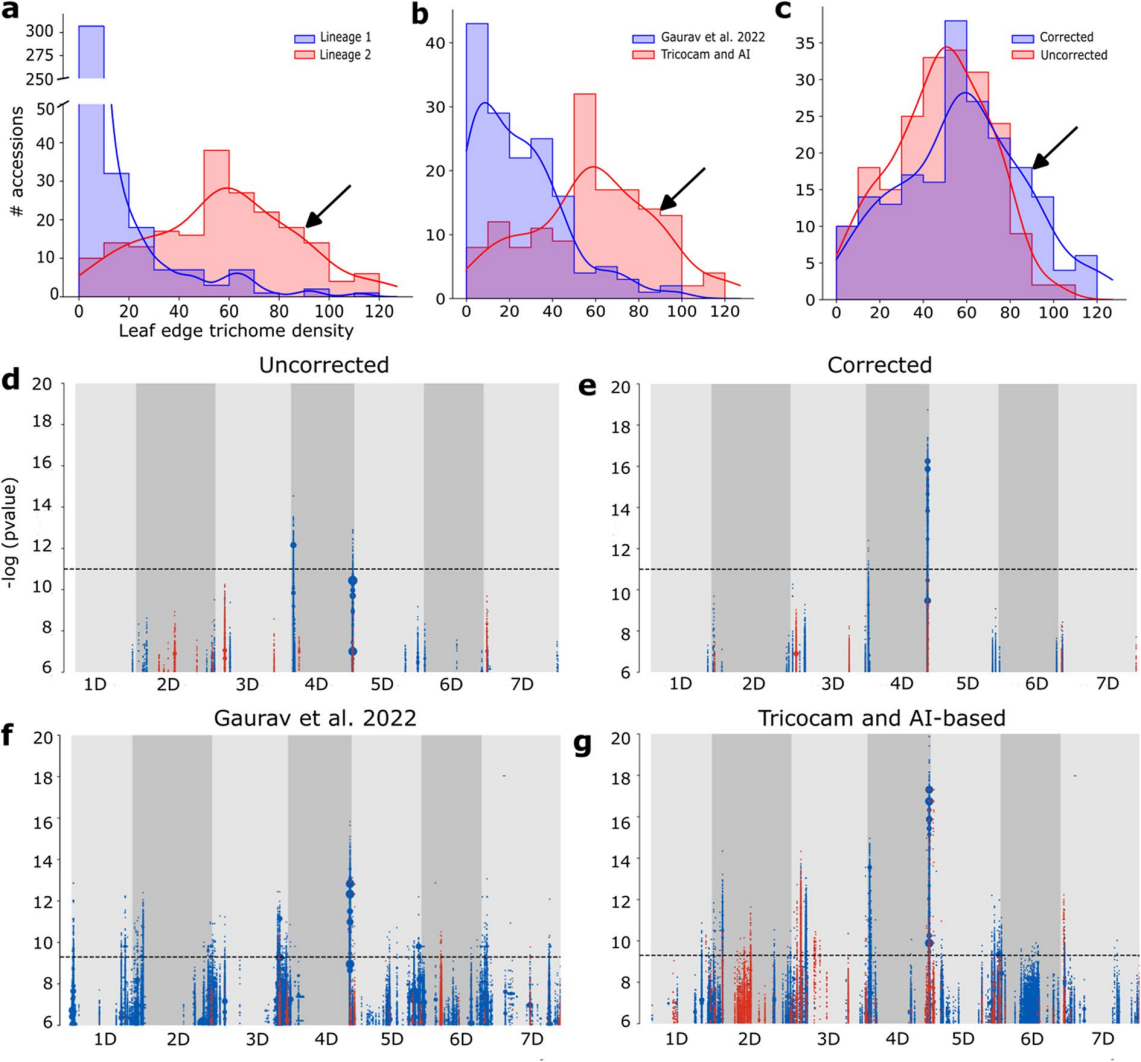
Leaf trichome density distribution in *Aegilops tauschii* and* k*-mer-based GWAS for association genetics. **a** Leaf edge trichome density distribution in 358 Lineage 1 and 203 Lineage 2 accessions. Scoring was done using the Tricocam and AI-based phenotyping system with manual correction. **b** Leaf edge trichome density distribution in datasets generated by Gaurav et al. (2022) and using the Tricocam and AI-based phenotyping system with manual correction. **c** Leaf edge trichome density distribution in 203 Lineage 2 accessions based on uncorrected and manually corrected datasets generated using the Tricocam and AI-based phenotyping system. **d** *k*-mer association mapping using uncorrected trichome density data for 140 non-redundant L2 accessions generated with the Tricocam and AI-based phenotyping system. **e** *k*-mer association mapping using manually corrected trichome density data for 140 non-redundant L2 accessions generated with the Tricocam and AI-based phenotyping system. **f** *k*-mer association mapping using trichome density data for 116 non-redundant L2 accessions taken from Gaurav et al. (2022). **g** *k*-mer association mapping using corrected trichome density data generated with Tricocam and AI-based phenotyping for the same set of 116 non-redundant L2 accessions used in Gaurav et al. (2022). For the Manhattan plots in d-g, the x-axis shows the seven chromosomes of the *Aegilops tauschii* genome and the y-axis shows the significance values of the associated *k*-mers mapped to the genome. Blue peaks indicate a positive correlation with the phenotype, while red peaks indicate a negative correlation. The horizontal dashed line indicates the significance threshold value based on Bonferroni correction. The association mapping was executed against the genome assembly of accession TOWWC0023, displaying high leaf edge trichome density (indicated by arrows in the histograms in a, b, and c)

Recently, a diversity panel of over 920 sequenced accessions of *Ae. tauschii*, including 493 genetically non-redundant (NR) accessions, and 46 high-quality genome assemblies was made available (Cavalet-Giorsa et al. 2024). We used this large-scale *Ae. tauschii* genomics resource to test our Tricocam high-throughput imaging and AI-based phenotyping system for gene discovery by quantifying leaf edge trichome density for the 616 accessions of this panel which were available to us (Supplementary Table 1; Fig. S1). Firstly, to visualize the phenotypic variation present in our population, we plotted the distribution of trichome density scores (Fig. 3a-c). We observed an effect of phylogenetic lineage on leaf edge trichome density (Fig. 3a). Among the panel, L1 accessions (n = 358) showed overall lower trichome density (mean 7.43 ± 15.60) compared to L2 accessions (n = 203) (mean 57.65 ± 28.58), while there were too few L3 accessions (*n* = 6) to make any meaningful comparison. We also compared our phenotypic data distribution for L2 accessions with phenotype data for 150 L2 accessions reported by Gaurav et al. (2022) (Fig. 3b). The 150 L2 from the previous study (Gaurav et al. 2022) were included among the 203 L2 accessions analyzed here (Fig. S1), thus highlighting the additional phenotypic diversity represented in the expanded panel of L2 accessions. Since the L2 accessions showed more variation alone than with the L1 accessions and no significant LD blocks were found using the L1 dataset (Fig. S3), we proceeded with the L2 dataset only for *k*GWAS. Additionally, we removed L2 accessions classified as genetically redundant (Gaurav et al. 2022; Cavalet-Giorsa et al. 2024) to reduce false positive associations, retaining 140 non-redundant L2 accessions (Fig. S1).

We estimated that 72% of the phenotypic variation of leaf edge trichome density in *Ae. tauschii* is attributed to genetic variation based on an estimated broad-sense heritability (H^2^) from 41 accessions grown in replicated experiments in three different controlled environments (Supplementary Table 2). We performed a type III ANOVA for genotypic and environmental effects and their interaction (Supplementary Table 2), observing significant effects of genotype F(40, 229) = 43.31, *p* =  < 2.2e-16, environment F(2, 229) = 73.6, *p* =  < 2.2e-16, and their interaction F(80, 229) = 2.74, *p* = 2.2e-09 on leaf edge trichome density.

We did not observe improvements to the quantification accuracy with the alternative YOLO-based detection model or augmentation of the images. Therefore, we assessed whether the accuracy levels obtained through training the un-augmented R-CNN model were sufficient for gene detection through GWAS, and to what degree correcting the dataset to reflect the “true” trichome number was able to improve the accuracy of gene detection. To obtain the true trichome number for each image, we took the annotated leaf images and manually corrected the detected number of trichomes by eye. This had the effect of shifting the distribution toward more accessions with > 80 trichomes per 20 mm (Fig. 3c).

We then used the uncorrected and corrected phenotype data for 140 non-redundant L2 accessions (Supplementary Table 1) to assess genotype–phenotype correlations using the *k*-mer-based GWAS (*k*GWAS) pipeline described by Gaurav et al. (2022) and the high-quality reference assembly for the L2 accession TOWWC0023 (Cavalet-Giorsa et al. 2024). This accession was scored as having an average of 92 trichomes per 20 mm, placing it in the upper 85th percentile of the L2 population (Fig. 3a–c). In both cases, we detected two significant trichome-controlling peaks, or linkage disequilibrium (LD) blocks, on chromosome arms 4DS and 4DL (Fig. 3d–g; Supplementary Table 3; Fig. S4). By comparing to the peaks obtained using the uncorrected dataset (Fig. 3d), the peak on 4DL increased ~ 500,000-fold in *k*GWAS significance after manual data correction, while the peak on chromosome arm 4DS was reduced in significance by ~ 100-fold (Fig. 3e). These results indicate that manual correction after automated quantification impacts association genetics and is necessary for more precise quantification.

Among the two LD blocks identified on chromosome 4D, we found that the association mapped to 4DS was novel compared to previous findings (Gaurav 2022; Mahjoob 2022; Dobrovolskaya 2007). By restricting the *k*GWAS to the same set of 116 non-redundant L2 accessions analyzed by Gaurav et al. (2022) and comparing to the *k*GWAS peaks obtained using that previous phenotype dataset (Fig. 3f), we observed that our phenotyping method alone was enough to yield the 4DS peak above the significance threshold and improve the power of the association mapping, increasing the significance of the 4DL peak ~ 1000-fold (Fig. 3g). Implementing our phenotyping method on the larger panel of 140 non-redundant L2 accessions further increased the significance of the major LD blocks on 4D compared to the smaller dataset of 116 non-redundant L2 accessions using the same Tricocam and AI-based method for both datasets (Fig. 3c, g). Overall, using the Tricocam device to phenotype *Ae. tauschii* leaf surface and analyzing a larger number of accessions both contributed to improving the association mapping for leaf trichome density in this wild wheat species.

## Discussion

There are several rapid plant phenotyping technologies available for a wide range of applications (Li et al. 2021; Gill et al. 2022; Xiao et al. 2022; Poorter et al. 2023). However, there is still potential for small-scale innovation and simple technology to further improve association genetics pipelines in crop species and their wild relatives. In this study, we leveraged the availability of 3D design and existing technology to produce the Tricocam which enabled rapid phenotypic data collection and improved the resolution of genotype–phenotype correlation in the wheat D genome progenitor species *Aegilops tauschii*. While replacement of the Faster-RCNN object detection algorithm with a YOLOv8 model and data augmentation did not improve accuracy of trichome counting, we showed the potential of this standardized imaging and AI-based quantification method to improve gene discovery.

Using our simple handheld device with an AI detection model and an easily observable phenotype, we reduced the time taken to collect and process phenotype data in a non-destructive manner for a large diversity panel of 610 accessions. Furthermore, our 3D print design requires minimal specialized training to replicate, and the trained AI detection model is available online. Such innovation can facilitate gene discovery using the abundance of genomic resources which are now available for many important crops (Li et al. 2014, 2024; Yang et al. 2023; Cheng et al. 2024; Jayakodi et al. 2024; Schreiber et al. 2024). We showed the potential of this standardized imaging and AI-based quantification method to improve association mapping for leaf trichome density in *Ae. tauschii*. We anticipate that high-throughput automated image capture and post-image processing will be more common in the future, allowing experimentalists to rapidly gather more data and associate traits with genotype. There are many applications in which smartphones and 3D printed devices, such as the Tricocam, can be used for image capture of other leaf traits of interest, including leaf area and shape for growth monitoring and drought response, leaf color for stress diagnosis and nutrient monitoring, detection of necrotic or chlorotic areas for disease phenotyping, vein patterning for taxonomy and water status monitoring, among other applications.

When applied to our panel of *Ae. tauschii* accessions, our Tricocam and AI-based phenotyping system uncovered a novel region of the *Aegilops tauschii* genome responsible for determining leaf edge trichome density. Additionally, by observing higher association significance with our phenotyping method we validated a genomic region that has been previously shown to be associated with this phenotype. Compared to previous studies that report marker-trait associations on chromosomes 2D, 3D, 4DL (QHl.ipk-4D), 5D, and 7D (Gaurav et al. 2022; Mahjoob et al. 2022; Dobrovolskaya et al. 2007), we identified an additional major locus underpinning trichome development on chromosome arm 4DS, thus highlighting this as a valuable pipeline for novel gene identification. While we reached the limits of accuracy for automatic detection of leaf edge trichomes with images taken by the Tricocam, this project presents the possibility for more automated phenotyping and image detection as a prequel to *k*GWAS and functional gene discovery in diversity panels of plant species.

Since orthologous LD blocks governing trichome formation have previously been found in different members of the Poaceae (CHang 1975; Dobrovolskaya et al. 2007; Luo et al. 2016; Saade et al. 2017; Wu et al. 2021), we hypothesize that these regions of the genome may retain a degree of conservation. Several QTLs have been reported across homeologous chromosomes in Triticeae species (Supplementary Table 4), suggesting the possibility of a shared genetic architecture of leaf trichome variation between some species, as exemplified by the homeologous group of barley 4HL *Hs*, rye 5RL *Hp1,* wheat 4BL/5AL, and *Ae. tauschii* 4DL QHl.ipk-4D locus (Dobrovolskaya et al. 2007; Korzun et al. 1999). Future inter-species comparisons using this phenotyping pipeline may reveal a conserved network of genes governing trichome development across species–genes that have been maintained through evolution. Any genes located within these LD blocks are likely to play a key role in controlling this agronomically important trait. Genes identified in this manner will also help elucidate the mechanism of trichome formation and function in grass species, which is not currently well understood.

Genes associated with tightly linked molecular markers can be used to track potentially useful or deleterious traits in breeding programs. *Ae. tauschii* has been the most used wild wheat species in wheat breeding programs. While much of the diversity from the A and B genome progenitors (i.e., *Triticum dicoccoides*) was captured during the hybridization that gave rise to hexaploid wheat, only about 25% of the genetic variation from the D genome progenitor (*Ae. tauschii*) was incorporated into bread wheat (Gaurav et al. 2022; Pont et al. 2019). Thus, to enhance D genome diversity in bread wheat, many breeders have returned to the progenitor species to create synthetic hexaploid wheats as part of pre-breeding programs (e.g., CIMMYT Global Wheat Program) (Gaurav et al. 2022; Rosyara et al. 2019; Zhou et al. 2021). In this context, deciphering the functional genetic framework underlying trichome formation across diverse grass species could ultimately pave the way for designing cereal crops with optimized trichome structures and plant-wide architectures, tailored for enhanced performance in varying environments while minimizing adverse effects on end-use quality.

## Supplementary Information

Below is the link to the electronic supplementary material.

Supplementary file1 (svg 5 kb)

Supplementary file2 (svg 4476 kb)

Supplementary file3 (ZIP 2948 kb)

Supplementary file4 (DOCX 2110 kb)

Supplementary file5 (XLSX 86 kb)
