# Correction: A high‑throughput pipeline for phenotyping, object detection and quantification of leaf trichomes

**DOI:** 10.1007/s00122-025-05035-2

**Published:** 2025-09-26

**Authors:** Andrea González‑Muñoz, Dai‑Jie Wu, Ana B. Perera‑Rodríguez, Mohamed Rekik, Silvio Giancola, Brande B. H. Wulff, Catherine Gardener

**Affiliations:** 1 Plant Science Program, Biological and Environmental Science and Engineering Division, King Abdullah University of Science and Technology (KAUST), Thuwal, Saudi Arabia; 2 Thya Technology, KAUST, Thuwal, Saudi Arabia

**Correction to: Theoretical and Applied Genetics (2025) 138:188** 10.1007/s00122-025-04967-z

In this published article, the Fig. 1 was in correctly published in the original publication. The complete correct Fig. 1 is given below.

**Fig. 1:**
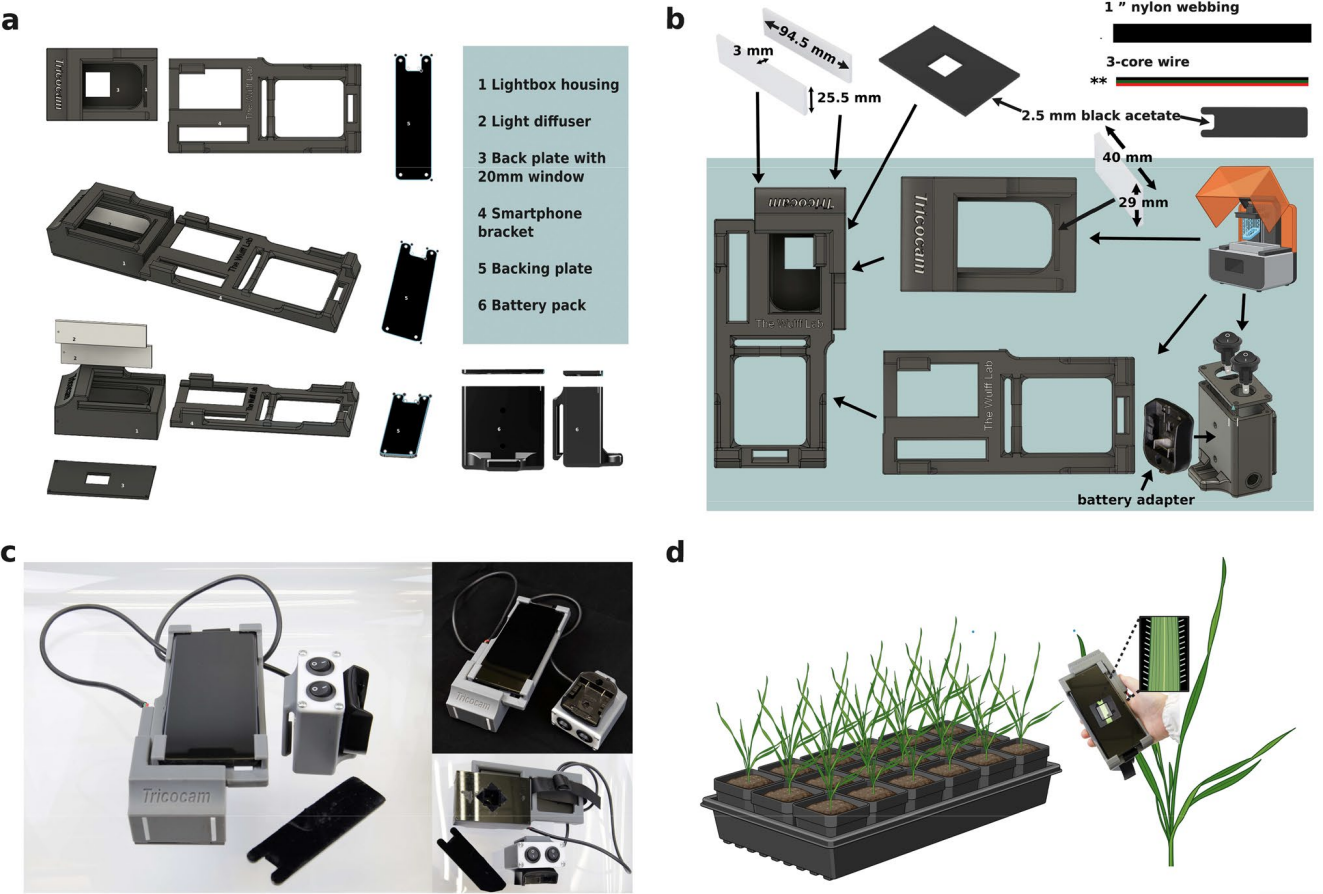
High-throughput and non-destructive leaf imaging in *Aegilops tauschii* using the Tricocam. **a** Diagram of device structure. **b** Tricocam assembly process. **c** Assembled Tricocam. **d** Phenotyping process with Tricocam

The original article has been corrected.

