# Supplementary material for: A high-throughput pipeline for phenotyping, object detection and quantification of leaf trichomes
Source: Theor Appl Genet. 2025 Jul 21;138(8):188. doi: 10.1007/s00122-025-04967-z (PMC12279567; doi:10.1007/s00122-025-04967-z)
Supplement: Supplementary file 4 — Supplementary file4 (DOCX 2110 kb) [file 122_2025_4967_MOESM4_ESM.docx]

**Supplementary Figures**


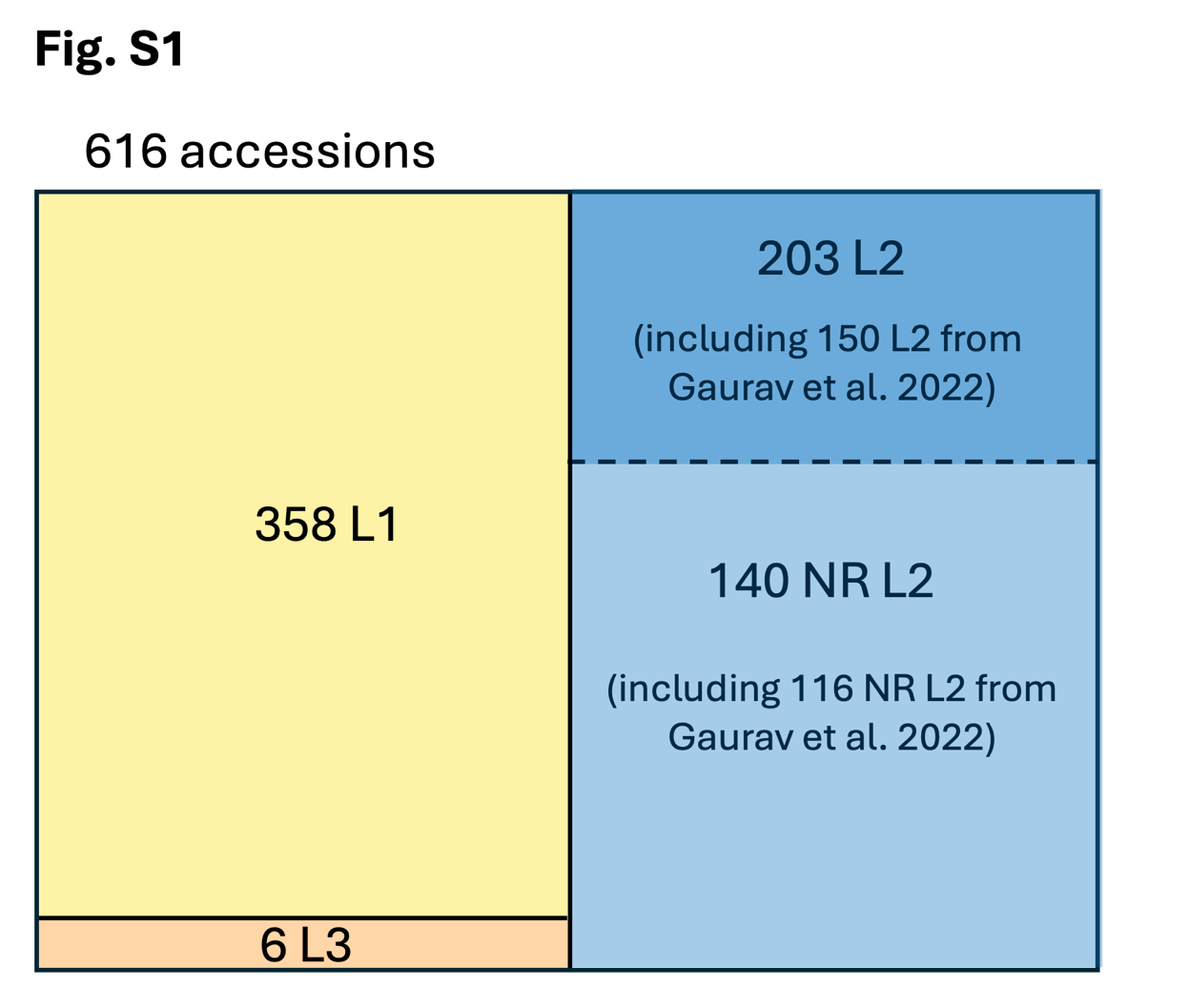


Set visualization of *Aegilops tauschii* accessions phenotyped using the Tricocam and trained trichome counting model. Out of the total 616 accessions, 358 were Lineage 1 (yellow inset), 203 Lineage 2 (blue inset) and 6 Lineage 3 (orange inset). Among the 203 Lineage 2 accessions, 150 accessions overlapped with the set characterized by Gaurav et al. (2022) (top dark blue inset). Out of the 203 Lineage 2 accessions, 140 were genetically non-redundant (NR) and used for *k*GWAS in this study (bottom light blue inset). This non-redundant L2 set included the 116 NR L2 accessions analyzed by Gaurav et al. (2022)

**
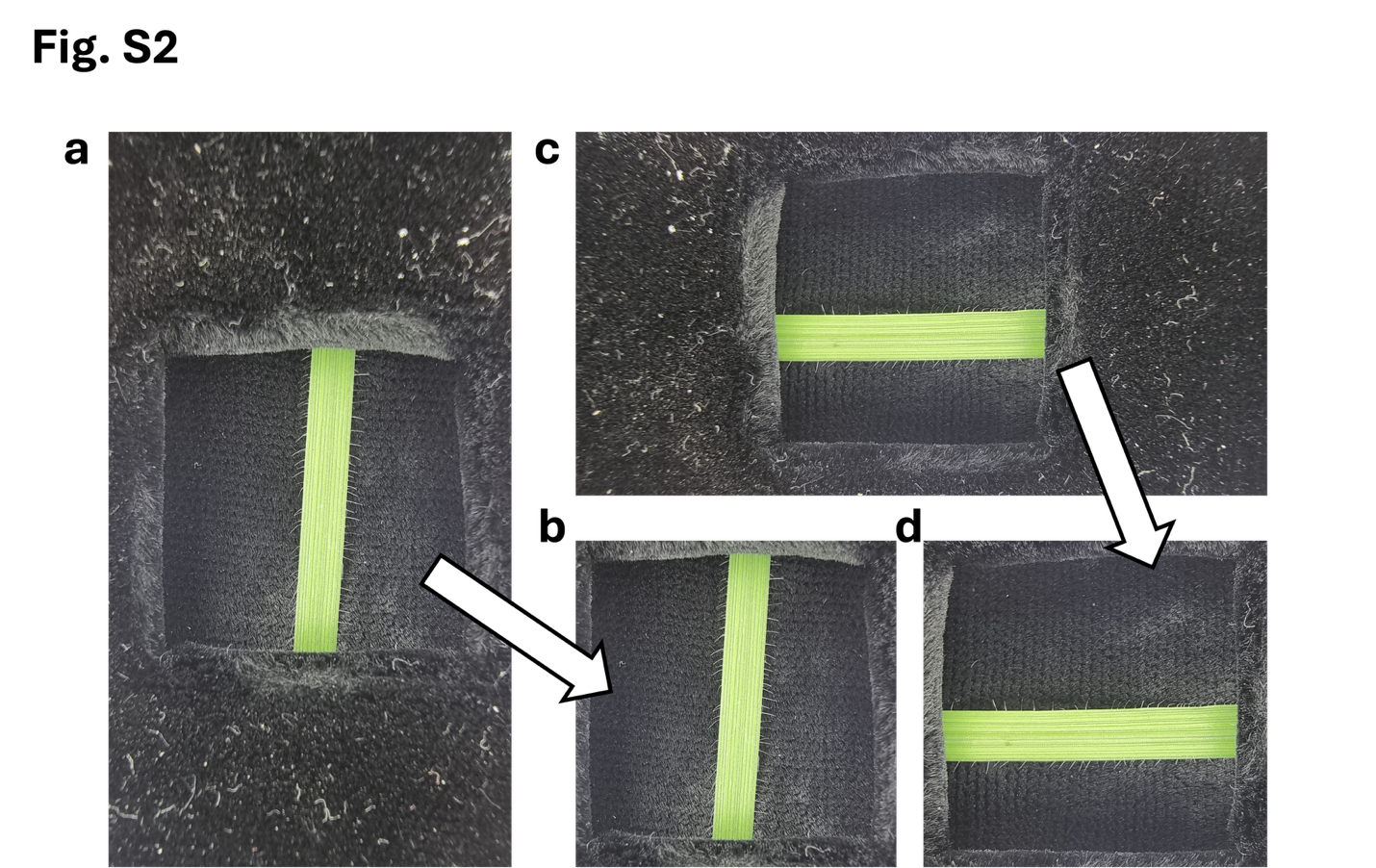
**

Image cropping process. An example of **a** a vertically orientated viewing window with a vertical oriented leaf, **b** the same image cropped to a square before the automated detection model is run. **c** a horizontally orientated viewing window with a horizontally orientated leaf, **d** the same image cropped before automated detection is performed


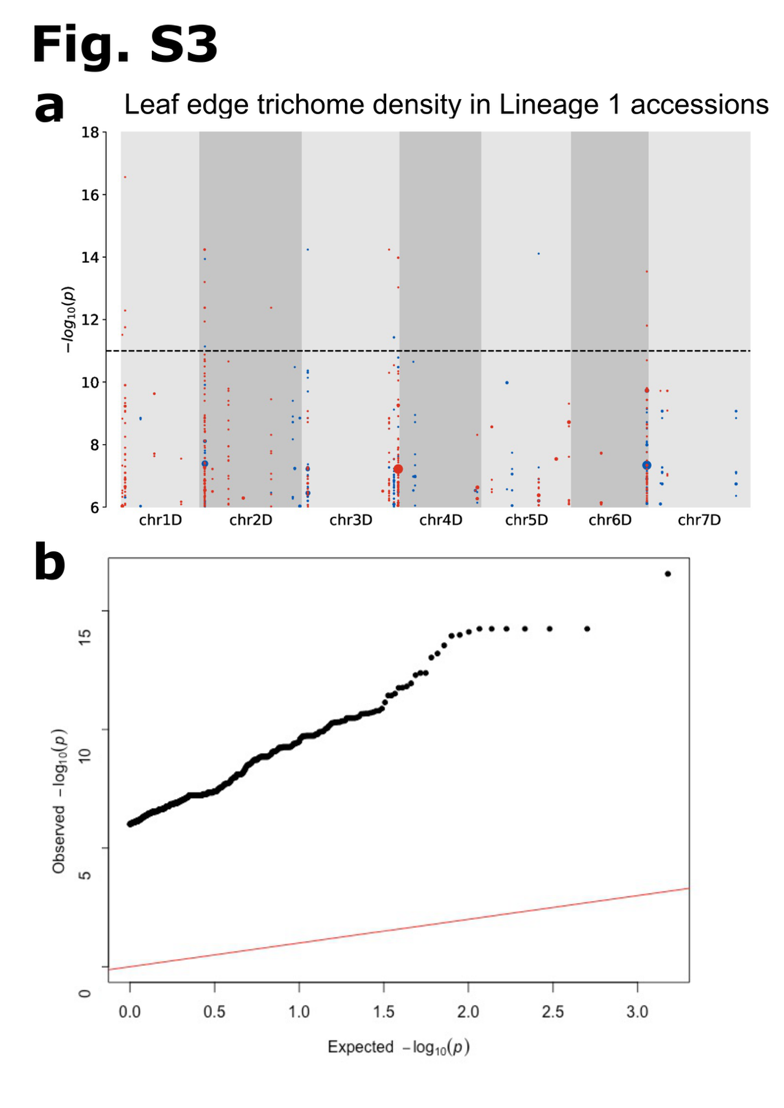


Association genetics for leaf edge trichome density in *Aegilops tauschii* Lineage 1 accessions. **a** *k*-mer association mapping using manually corrected trichome density data for 335 non-redundant Lineage 1 accessions generated with the Tricocam and AI-based phenotyping system. **b** Q-Q plot of -log_10_(pvalues) for the significantly associated *k*-mers shown in the Manhattan plot in panel a


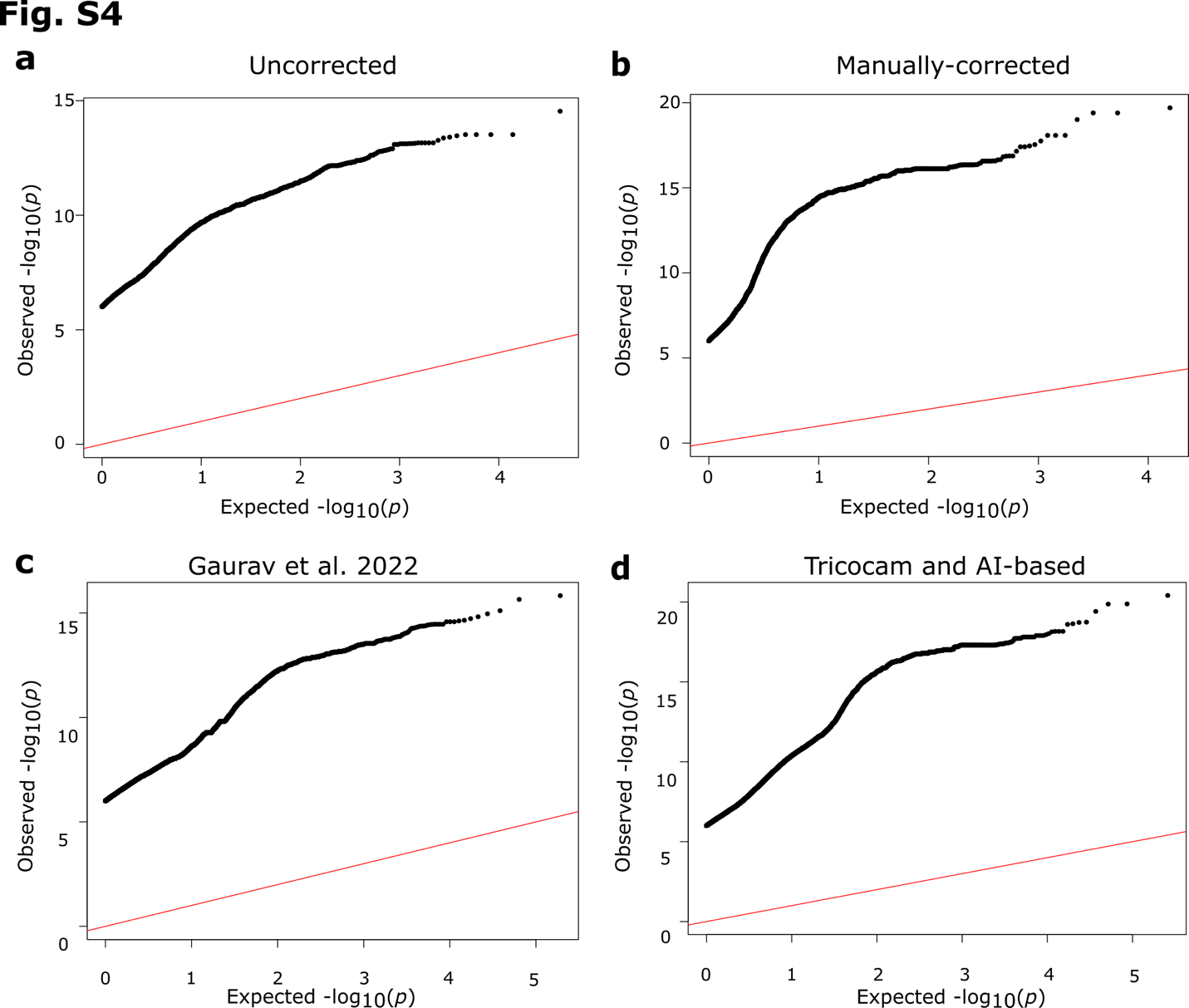


Q-Q plots of -log_10_(pvalues) for the significantly associated *k*-mers obtained from *k*GWAS for leaf edge trichome density in *Aegilops tauschii* using different phenotype datasets: **a** uncorrected trichome density data for 140 non-redundant L2 accessions generated with the Tricocam and AI-based phenotyping system. **b** manually corrected trichome density data for 140 non-redundant L2 accessions generated with the Tricocam and AI-based phenotyping system. **c** trichome density data for 116 non-redundant L2 accessions taken from Gaurav et al. (2022). **d** corrected trichome density data generated with Tricocam and AI-based phenotyping for the same set of 116 non-redundant L2 accessions used in Gaurav et al. (2022)
